# Cholera Prevention

**Published:** 1890-08-30

**Authors:** 


### The Doctor's Pulpit.

### CHOLERA PREVENTION.

Cholera is a disease of the East rather than of the West; but especially it is a disease which prevails most extensively and fatally in ill-drained and filthy towns, and among careless and dirty people. India appears to be its native abode; and if eminent medical writers may be relied upon, that country has given rise to every historical epidemic in whatever part of the world it may have prevailed. "The disease has never broken out spontaneously in any other part of the world; no amount of filth, bad food, or climatic influences have, up to the present time, induced a widespread epidemic of cholera," says Mr. Macnamara in Quain's *Dictionary of Medicine.*

What is cholera? What is the cause of it? How is it to be prevented? Clear and simple answers to these questions may be of great service at the present time. Cholera in its widest meaning may range between simple diarrhœa on the one hand, and a prostrating attack of vomiting and purging on the other. But the cholera that is thoroughly alarming, and which all the resources of civilization should be invoked to prevent, is the Asiatic variety. What, then, constitutes true Asiatic cholera? There are four conditions which usually manifest themselves in a typical case; these are severe vomiting, excessive purging with rice water evacuations, painful cramps in the stomach and legs, and profound prostration. In the worst cases all these symptoms will be markedly pronounced. When the attack is milder one or more of them may be diminished in severity. There is no need to enlarge upon the danger of such a seizure as we have described. Any reader who has ever experienced a very serious attack of vomiting alone, or of diarrhœa alone, can readily imagine what the two together must be when each of them is of a violent and excessive kind. When in addition there are cramps in both stomach and legs of the most painfully distressing and persistent character, there is no difficulty in picturing the profound collapse and the imminent risk of death which must necessarily result. Of course the four leading symptoms just mentioned do not by any means exhaust the peculiar appearances of a typical case of Asiatic cholera, but they are sufficient for the practical purpose of enabling any intelligent person to make a shrewd guess at the nature of the disease when he sees it.

The second question we propose to answer briefly is, What is the cause of cholera? From the point of view of persons who rely upon prevention this is more important than our first question. The best medical science of our times affirms that the cause of cholera is always and everywhere one and the same. A true and proper case of Asiatic cholera can only be produced by the entrance of the true and proper cholera bacillus into the blood of a living person; and this is so in the same sense that a field of wheat can be produced in no other way but by the previous sowing of seed wheat in the ground. If a fallow field be left to itself it will sooner or later produce a great variety of plants which we call weeds, and that for the very simple reason that the seeds of such weeds are always in the ground, never having been got rid of by cultivation. But the fallow will not produce a crop of wheat unless the seed of wheat have been previously brought to the field and sown in it. According to present-day medical science the commencement and development of a case of cholera is exactly similar to the commencement and development of a crop of standing wheat.

The question then for the practical preventor is this —Where does the seed bacillus come from which produces an epidemic of Asiatic cholera? It comes from existing bacillus crops, exactly as seed wheat comes from wheat crops. It cannot originate in any other way. A patient attacked by Asiatic cholera vomits and is violently purged. The evacuations are received into a vessel; the vessel then contains the cholera bacillus, the seed of future crops. If a smaller or larger number of the bacilli contained in the vessel enter the blood of a healthy person they will, under favouring circumstances, set up cholera in that person within a very short time. But how can the bacilli in the vessel find their way into the blood of a healthy person? In one way, certainly; perhaps in two or more. The vessel is in all probability emptied into the drain; if the drain has any communication, however slight, with the source of the supply of drinking water, some of the contents of the vessel may find their way into the drinking water and be swallowed. In such a case an attack of Asiatic cholera will almost certainly follow. But sometimes the vessel containing the evacuations is left uncovered in the room of the sick person for a time, or carried along lobbies and passages, or placed all exposed in some other room and left there. The bacilli seem to have power to separate themselves from the evacuations, and to float up in the air like motes which a sunbeam renders visible. The air, charged with bacilli, may then be inhaled, and passing into the lungs, may give up its bacilli to the lungs just as it gives up its oxygen. In such a case also an attack of cholera will almost certainly follow. The important point to bear in mind is that the bacilli are present principally, if not entirely, in the patient's evacuations. They do not seem to be given off by the breath or by the skin, and therefore there is little or no risk in being present in the room with a cholera patient, or in touching him, or rendering him necessary services; always provided, of course, that the evacuations are received into an aseptic vessel, and at once disposed of aseptically. To repeat, the one only cause of dangerous cholera is the cholera bacillus, and that is not dangerous to healthy persons, unless by some means or other it finds its way into the blood.

How then is cholera to be prevented? All intelligent persons who have definitely grasped the principles contained in the preceding statements, will see at once how prevention is to be effected. The evacuations are the sources of the danger; then the evacuations must, if possible, be rendered harmless, and they must be removed to a place of safety with all possible promptitude. This is the great secret of the prevention of the spread of cholera. Of course it must be borne in mind that whilst it is essential to remove the evacuations from the neighbourhood of healthy persons, it is equally desirable to remove healthy persons from the neighbourhood of the evacuations. In other words, when a person is attacked with Asiatic cholera, he should be immediately isolated, either by being taken at once to a hospital, or by the prompt departure of all, except the necessary attendants, from the house where he happens to be living. The three chief things to be borne in mind, then, in case of an outbreak of cholera are isolation, the thorough disinfection and destruction of all the patient's evacuations, and the most scrupulous cleanliness in every part of the building and neighbourhood where he is placed. A simple method of disinfecting evacuations is that of having two or three vessels, each containing a small quantity of fluid disinfectant, always ready for use. After one of the vessels has been used the contents should also be entirely covered with a disinfectant. Mr. Macnamara recommends bichromate of potash, or Macdougall's mixture for the bottom of the vessel, and advises that carbolic acid in the proportion of one part to twenty of water should be sprinkled or poured upon the dejecta. The contents of the vessels thus treated may be disposed of by being buried deeply in the earth, but care should be taken that they are placed at a safe distance from wells and running water. If, as in large towns, there is no convenience for thus disposing of them, they may be thrown down the drains, provided, however, that there is a constant supply of fresh water available for flushing. If the drains are to be used, a solution of sulphate of iron in the proportion of an ounce of the sulphate to a pint of water should be poured freely into them after they have been well flushed. There are other disinfectants which will serve the purpose as well as those we have named, but it is necessary to bear in mind that substances used for disinfecting vary greatly in strength and activity. When cholera is active, disinfectants of the most powerful kind should alone be employed. There is some reason to fear that cholera may visit our shores at no distant date. To prevent such a calamity, public bodies and private persons must do all that is possible to cleanse and purify houses, streets, and especially drains. If, in spite of such measures, the dreaded enemy should still attack us, we may feel assured that the facts which science has made known to us, and the simple measures indicated, will, if properly carried out, enable us to give a thoroughly good account of ourselves.

